# Investigating mitochondrial bioenergetics in peripheral blood mononuclear cells of women with childhood maltreatment from post-parturition period to one-year follow-up

**DOI:** 10.1017/S0033291722000411

**Published:** 2023-07

**Authors:** Anja M. Gumpp, Alexander Behnke, Laura Ramo-Fernández, Peter Radermacher, Harald Gündel, Ute Ziegenhain, Alexander Karabatsiakis, Iris-Tatjana Kolassa

**Affiliations:** 1 Clinical & Biological Psychology, Institute of Psychology and Education, Ulm University, Ulm, Germany; 2 Institute of Anesthesiological Pathophysiology and Process Engineering, University Hospital Ulm, Ulm, Germany; 3 Department of Psychosomatic Medicine and Psychotherapy, University Hospital Ulm, Ulm, Germany; 4 Department of Child and Adolescent Psychiatry and Psychotherapy, University Hospital Ulm, Ulm, Germany; 5 Clinical Psychology, Institute of Psychology, University of Innsbruck, Innsbruck, Austria

**Keywords:** Bioenergetics, childhood maltreatment, mitochondria, mitochondrial density, PBMC, postpartum

## Abstract

**Background:**

Childhood maltreatment (CM) exerts various long-lasting psychological and biological changes in affected individuals, with inflammation being an interconnecting element. Besides chronic low-grade inflammation, CM might also affect the energy production of cells by altering the function and density of mitochondria, i.e. the body's main energy suppliers. Here, we compared mitochondrial respiration and density in intact peripheral blood mononuclear cells (PBMC), from women with and without CM between two time points, i.e. at the highly inflammatory phase within 1 week after parturition (*t*_0_) and again after 1 year (*t*_2_).

**Methods:**

CM exposure was assessed with the *Childhood Trauma Questionnaire*. Whole blood was collected from *n* = 52 healthy women within the study ‘My Childhood – Your Childhood’ at both time points to isolate and cryopreserve PBMC. Thawed PBMC were used to measure mitochondrial respiration and density by high-resolution respirometry followed by spectrophotometric analyses of *citrate-synthase* activity.

**Results:**

Over time, quantitative respiratory parameters increased, while qualitative flux control ratios decreased, independently of CM. Women with CM showed higher mitochondrial respiration and density at *t*_0_, but not at *t*_2_. We found significant CM group × time interaction effects for *ATP-turnover*-related respiration and mitochondrial density.

**Conclusions:**

This is the first study to longitudinally investigate mitochondrial bioenergetics in postpartum women with and without CM. Our results indicate that CM-related mitochondrial alterations reflect allostatic load, probably due to higher inflammatory states during parturition, which normalize later. However, later inflammatory states might moderate the vulnerability for a second-hit on the level of mitochondrial bioenergetics, at least in immune cells.

## Introduction

Childhood maltreatment (CM) includes experiences of physical, sexual, and/or emotional abuse, as well as physical and/or emotional neglect during childhood and adolescence. Experiences of CM present aversive, often chronic, and repetitive stressors (WHO, 2020) that can have lifelong detrimental health consequences. Individuals with CM have a higher risk to develop somatic (e.g. cardiovascular or metabolic diseases) and mental diseases (e.g. depression or posttraumatic stress disorder) later in life (Nemeroff, 2016).

Previous research linked the elevated physical and mental morbidity in individuals with CM to profound and persistent alterations in the psychobiological reactivity to stress, assuming a dysregulation of the hypothalamic-pituitary-adrenal (HPA) axis (reviewed in Guilliams & Edwards, 2010; Tarullo & Gunnar, 2006) along with persistent immunological alterations involving chronic low-grade inflammation and elevated levels of oxidative stress (Boeck et al., 2016; Coelho, Viola, Walss-Bass, Brietzke, & Grassi-Oliveira, 2014; Danese, Pariante, Caspi, Taylor, & Poulton, 2007; do Prado et al., 2016). A common mechanism behind these alterations could be changes in mitochondrial bioenergetics. Mitochondria are not only the essential provider of cellular energy for stress and immune responses, they are also the main source of oxidative stress and are viewed as ‘sovereign of inflammation’ (Mills, Kelly, & O'Neill, 2017; Tschopp, 2011).

Mitochondria are highly dynamic organelles found in almost all eukaryotic cells to which they provide cellular energy in the form of adenosine triphosphate (ATP). Through a process called oxidative phosphorylation (OXPHOS), mitochondria use oxygen to gradually degrade nutrients (e.g. sugars, amino acids, and fatty acids) to produce ATP (Wilson, 2017). Additionally, mitochondria are important regulators of inflammatory processes (Nakahira et al., 2011; Sorbara & Girardin, 2011; Zhou, Yazdi, Menu, & Tschopp, 2011) through the emission of reactive oxygen species (ROS) (López-Armada, Riveiro-Naveira, Vaamonde-García, & Valcárcel-Ares, 2013; Valko et al., 2007) and the secretion of mitochondrial DNA (mtDNA) as inflammatory signals (Hummel et al., 2018; Trumpff et al., 2019). They are further involved in the regulation of cellular calcium homeostasis (Pizzo, Drago, Filadi, & Pozzan, 2012), redox balance (Schulz, Wenzel, Münzel, & Daiber, 2014), and apoptosis (Newmeyer & Ferguson-Miller, 2003).

Recent research linked alterations in mitochondrial bioenergetics to various adverse mental and physical health conditions (Daniels, Olsen, & Tyrka, 2020; Srivastava, 2017). For example, changes in mitochondrial bioenergetics were observed in major depression as a highly prevalent mental health consequence of CM (Gumpp et al., 2021; Hroudová, Fišar, Kitzlerová, Zvěřová, & Raboch, 2013; Karabatsiakis et al., 2014; Kuffner et al., 2020).

There is also growing evidence that a history of CM might exert effects on mitochondrial bioenergetics in adulthood. In previous studies, we demonstrated that postpartum women with CM experiences showed elevated levels of mitochondrial function in peripheral blood mononuclear cells (PBMC) (Boeck et al., 2016; Gumpp et al., 2020). We found elevated basal mitochondrial activity, increased *ATP-turnover*-related respiration, and higher mitochondrial density in women with CM shortly after parturition (Gumpp et al., 2020). Mitochondria of women with CM at 3 months postpartum still exhibited higher levels of basal mitochondrial activity and increased *ATP production*-related respiration compared to women without CM. However, there were no differences anymore with respect to intracellular mitochondrial density as measured through the activity of the mitochondrial enzyme Citrate synthase (Boeck et al., 2016).

Upregulated mitochondrial activity and density following parturition might reflect the elevated energy demand of pregnancy and parturition itself (King, 2000) as well as the increased inflammatory activity due to postpartum wound healing (Christian & Porter, 2014; Corwin, Bozoky, Pugh, & Johnston, 2003; Maes, Ombelet, De Jongh, Kenis, & Bosmans, 2001; Stewart et al., 2007). We accordingly showed among postpartum women that the increased mitochondrial activity was positively linked to the spontaneous release of pro-inflammatory cytokines by PBMCs (Boeck et al., 2016). Chronic psychological stress such as CM negatively impacts the detrimental effects on the organism's capability of self-repair and immunity (Dhabhar, 2014) and compromises the physiological processes of wound healing (Christian, Graham, Padgett, Glaser, & Kiecolt-Glaser, 2006; Kiecolt-Glaser, Marucha, Mercado, Malarkey, & Glaser, 1995). Women with a CM history might thus present increased inflammatory activity and, presumably, slower wound healing in the time between parturition and the immunological ‘recovery’ tothe pre-pregnant immune state which may take up to 1 year (Watanabe et al., 1997). Accordingly, upregulated mitochondrial activity and density following parturition could be a transient state limited to the first months after giving birth. However, no study has yet examined the temporal stability of CM-associated changes in mitochondrial bioenergetics and biogenesis across 1 year postpartum.

However, there is also the first evidence that CM-related mitochondrial alterations might persist beyond phases of an acute physiological challenge. Tyrka et al. (2016) demonstrated higher mtDNA copy number (mtDNAcn) as another marker of mitochondrial content in leukocytes of non-pregnant, non-postpartum adults with CM compared to those without CM, indicating that CM-related alterations in mitochondrial density might not be limited to the postpartum period. Another study also reported significantly higher mtDNAcn being linked to experiences of childhood sexual abuse in clinically depressed women; however, this finding did not translate to healthy controls (Cai et al., 2015).

Based on the aforementioned findings, we investigate whether (i) the higher mitochondrial respiratory activity and density observed in mothers with CM compared to mothers without CM shortly occurs specifically after parturition, presenting an ‘allostatic state’ due to the burden of postpartum inflammation and wound healing; or (ii) whether it represents a permanent (‘trait’-like) alteration that is present beyond the postpartum phase. For this purpose, we analyzed data from the longitudinal study ‘My Childhood – Your Childhood’ to characterize the mitochondrial bioenergetic profile in PBMCs of healthy women with and without CM at 1 week after parturition and at 1 year later.

## Methods and materials

### Study design

Participants consisted of healthy community women who were recruited within the longitudinal study ‘My Childhood – Your Childhood’ from October 2013 to December 2015 (for more details see Koenig et al., 2018 and online Supplementary Fig. S1). In short, mothers were recruited within 1 week after parturition [*M* (s.d.) = 2.3 (1.2) days for the cohort of this paper]. Exclusion criteria for mothers were insufficient knowledge of the German language, severe complications during parturition (e.g. stillbirths), any severe health problems of either mother or child (e.g. admission of the newborns or mothers to the intensive care unit), and maternal age under 18 years (see online Supplementary Fig. S1). Dropout rates are reported in online Supplementary Fig. S1.

After obtaining written informed consent, sociodemographic and clinical data were assessed in a psychodiagnostic interview (*t*_0_). The German short version of the *Childhood Trauma Questionnaire* (CTQ; Bader, Hänny, Schäfer, Neuckel, & Kuhl, 2009; Bernstein & Fink, 1998) was used to assess retrospectively adverse childhood experiences (abuse and neglect). Trained psychologists conducted the CTQ as an interview due to the emotionally sensitive situation of the participating women. Using standardized and established mild cut-off criteria for the CTQ, women without any CM experiences were classified as CM−, whereas those with at least mild CM experiences in at least one CTQ subscale were categorized as CM+ (Bernstein & Fink, 1998). The CTQ sum score (possible range 25–125) was used as a cumulative burden of CM exposure (Schury & Kolassa, 2012).

At the 3 months postpartum follow-up (*t*_1_), current and lifetime psychiatric disorders of the women were examined using the German research version of the *Structured Clinical Interview* (*SCID-I*) (Wittchen, Zaudig, & Fydrich, 1997) for the diagnosis of axis-I disorders according to the Diagnostic and Statistical Manual of Mental Disorders, version IV (DSM-IV-TR; American Psychiatric Association, 2000). The SCID-I has been updated by experienced clinicians to allow for diagnosis according to the Diagnostic and Statistical Manual of Mental Disorders, version 5 (DSM-5; American Psychiatric Association, 2013).

About 1 year after parturition [*M* (s.d.) = 381.7 (28.6) days for the cohort of this paper], the women were re-invited for a second follow-up (*t*_2_; for drop-out rates see online Supplementary Fig. S1) comprising a psychological interview and assessments of sociodemographic and medical data. Participants received a small remuneration each time they participated in the study.

Mothers with and without CM only differed in the CTQ sum score and the CTQ subscale scores, but not in other descriptive characteristics (Table 1). All study procedures were approved by the Ethics Committee of Ulm University and conducted in accordance with the Declaration of Helsinki (World Medical Association, 2013).

**Table 1.** Sociodemographic and clinical characteristics of the women (*n* = 52)

	CM− group (*n* = 29) *M* [s.d.] or *n* (%)	CM+ group (*n* = 23) *M* [s.d.] or *n* (%)	Test statistics^1^	*p*
*Sociodemographics*				
Maternal age at *t*_0_ (years)	33 [4]	33 [5]	*t*(*50*) = −0.70, *d* = −0.20	0.485
Time between childbirth and *t*_2_ (days)	383 [32]	380 [25]	*t*(*50*) *=* *−*0.43, *d* = −0.12	0.672
BMI at *t*_2_ (kg/m^2^)^2^	24.0 [3.4]	23.7 [4.8]	*U* = 774.00, *r* = −0.10	0.498
Smoking during pregnancy	2 (7%)	2 (9%)	χ^2^(1)<0.001, *V* < 0.001	1.000
Smoking at *t*_2_	2 (7%)	3 (13%)	χ^2^(1) = 0.08, *V* = 0.04	0.644
Maternal ethnicity [Caucasian]	29 (100%)	23 (100%)	–	–
Living in a partnership (at *t*_0_ and *t*_2_)	29 (100%)	23 (100%)	–	–
Academic education	23 (79%)	13 (57%)	χ^2^(1) = 2.15, *V* = 0.20	0.143
Number of children	2 [1]	2 [1]	*U* = 727.5, *r* = 0.10	0.395
Pregnancy duration (days)	280 [7]	278 [8]	*t*(*50*) *=* *−*0.84, *d* = −0.23	0.406
Vaginal delivery^3^	20 (71%)	20 (87%)	χ^2^(1) = 1.00, *V* = 0.14	0.305
*Psychological & clinical characteristics*				
Lifetime psychiatric disorder (SCID-I)^4^	8 (29%)	11 (50%)	χ^2^(1) = 1.58, *V* = 0.18	0.209
Major depression^4^	4 (14%)	8 (36%)	χ^2^(1) = 2.19, *V* = 0.21	0.099
Anxiety disorder^4^	4 (14%)	5 (23%)	χ^2^(1) = 0.16, *V* = 0.06	0.482
Lifetime intake of psychotropic medication	8 (29%)	7 (30%)	χ^2^(1)<0.001, *V* < 0.001	1.000
Intake of psychotropic medication since childbirth at *t*_2_^5^	2 (7%)	1 (5%)	χ^2^(1)<0.001, *V* < 0.001	1.000
CTQ sum score	27.3 [1.9]	39.8 [12.1]	*U* = 456.50, *r* = 0.80	<0.001***
Emotional abuse sum score	5.7 [0.9]	8.5 [3.9]	*U* = 589.50, *r* = 0.48	<0.001***
Physical abuse sum score	5.1 [0.3]	6.3 [2.2]	*U* = 669.50, *r* = 0.37	0.008**
Sexual abuse sum score	5.0 [0]	7.3 [4.4]	*U* = 652.50, *r* = 0.47	<0.001***
Emotional neglect sum score	6.5 [1.5]	11.5 [4.1]	*U* = 528.00, *r* = 0.62	<0.001***
Physical neglect sum score	5.1 [0.3]	6.3 [2.4]	*U* = 684.00, *r* *=* 0.33	0.018*

Note. *p < 0.050, **p < 0.010, ***p < 0.001, two-tailed. CM, Childhood maltreatment; CM+, Women with at least mild to severe CM experiences; CM−, Women without CM experiences; CTQ, Childhood Trauma Questionnaire.

1 Two-tailed Student's *t* tests/Mann–Whitney *U* tests/χ^2^-tests/Fisher's exact tests were calculated where appropriate. CM− group as a reference group. As effect size measure Cohen's *d*, *r*, or Cramer's *V* is reported.

2 *n* = 50, two missings in CM+ group.

3 *n* = 51, one missing in CM− group.

4 SCID-I was assessed at 3 months postpartum (*t*_1_). SCID-I data are available for *n* = 50 women (CM− group: *n* = 28, CM+ group: *n* = 22); multiple diagnoses are possible; additional lifetime diagnoses: specific phobia (*n* *=* 4), panic disorder (*n* = 3), alcohol disorder (*n* = 2), acute stress disorder (*n* = 1), adjustment disorder (*n* = 1), agoraphobia (*n* = 1), posttraumatic stress disorder (*n* = 1), social phobia (*n* = 1), unspecific anxiety disorder (*n* = 1).

5 *n* = 51, one missing in CM+ group.

### Peripheral blood processing

At *t*_0_ and *t*_2_, peripheral non-fasting venous blood from the women (up to 32 ml) was drawn into Citrate-phosphate-dextrose-adenine (CPDA)-buffered monovettes (Sarstedt, Nürmbrecht, Germany). PBMC were isolated by Ficoll-Hypaque density gradient centrifugation (GE Healthcare, Chalfont St Giles, UK) according to the manufacturer's protocol and stored frozen at −80 °C in cryopreservation medium (dimethyl sulphoxide: Sigma-Aldrich, St. Louis, MO, USA; fetal calf serum (FCS): Sigma-Aldrich; dilution 1:10) until biological analyses. Additionally, a small volume of venous blood was collected into EDTA-buffered blood monovettes (Sarstedt) at both time points to generate standard hemograms at the Institute for Clinical Chemistry of Ulm University to account for possible changes in the blood cell composition. Blood cell compositions of postpartum women at *t*_0_ (Gennaro et al., 1997) and *t*_2_ (Perkins, 1999) were comparable to other studies. CM− and CM+ mothers did not differ in hemograms (blood cell composition; see Table 2) at *t*_0_ (leukocytes: *U* = 700.5, *r* = −0.17, *p* = 0.258; lymphocytes: *t*(45) *=* 0.90, *d* = 0.26, *p* = 0.374; monocytes: *t*(45) *=* *−*0.39, *d* = −0.12, *p* = 0.696) or at *t*_2_ (leukocytes: *U* = 723.5, *r* = −0.03, *p* = 0.853; lymphocytes: *t*(48) = 1.12, *d* *=* 0.32, *p* = 0.269; monocytes: *t*(48) *=* *−*0.23, *d* = −0.06, *p* = 0.822).

**Table 2.** Biological raw data of the women at *t*_0_ and *t*_2_ (*n* = 52)

	*t*_0_ (*n* = 52)		*t*_2_ (*n* = 52)	
	CM− group (*n* = 29) *M* [s.d.]	CM+ group (*n* = 23) *M* [s.d.]	CM− group (*n* = 29) *M* [s.d.]	CM+ group (*n* = 23) *M* [s.d.]
Primary mitochondrial respiration parameters (pmol O_2_/sec per Mio cells)				
Routine respiration	3.21 [0.65]	3.57 [0.60]	3.17 [0.54]	3.24 [0.36]
Leak respiration	0.95 [0.29]	1.07 [0.24]	1.21 [0.23]	1.30 [0.16]
ATP-turnover-related respiration	2.27 [0.50]	2.50 [0.47]	1.96 [0.48]	1.95 [0.34]
Uncoupled respiration	5.66 [1.32]	6.54 [1.13]	6.61 [1.20]	6.94 [1.41]
Spare respiratory capacity	2.45 [1.15]	2.97 [1.02]	3.44 [0.90]	3.69 [1.18]
Residual oxygen consumption (ROX)	0.28 [0.22]	0.20 [0.18]	0.11 [0.05]	0.11 [0.05]
Mitochondrial density (pmol/sec per Mio cells)				
*Citrate-synthase* activity	62.36 [8.93]	70.97 [12.28]	68.72 [8.86]	68.47 [9.25]
Flux control ratios (%)				
Routine control ratio	58 [13]	56 [11]	48 [6]	48 [8]
Leak control ratio	17 [5]	17 [5]	19 [5]	19 [5]
Net routine ratio	41 [10]	39 [7]	30 [4]	29 [4]
Coupling efficiency	71 [6]	70 [5]	61 [7]	60 [5]
Blood cell composition^1^				
Leukocytes (×10^3^/*μ*l)	11.4 [3.2]	10.3 [2.3]	7.1 [1.6]	7.0 [1.5]
Lymphocytes (% in whole blood)	18.2 [4.3]	19.3 [4.3]	29.6 [6.2]	31.6 [5.9]
Monocytes (% in whole blood)	5.8 [1.6]	5.6 [1.3]	6.8 [1.8]	6.7 [1.1]
Storage time of cryopreserved cells				
Duration of cell cryopreservation (days)^2^	1079 [220]	1146 [242]	1218 [167]	1216 [202]

CM, childhood maltreatment; CM+, Women with at least mild to severe CM experiences; CM−, Women without CM experiences.

All measures are given as mean [standard deviation]. Primary mitochondrial respiration parameters are presented corrected for Residual oxygen consumption (ROX). For the range (minimum-maximum) see online Supplementary Table S1.

1 Blood counts available from *n* = 47 women at *t*_0_ (CM− group: *n* = 27, CM+ group: *n* = 20) and *n* = 50 women at *t*_2_ (CM− group: *n* = 28, CM+ group: *n* = 22); Blood cell compositions of postpartum women at *t*_0_ (Gennaro et al., 1997) and at *t*_2_ (Perkins, 1999) were comparable to other studies. CM− and CM+ mothers did not differ in blood cell composition at *t*_0_ (leukocytes: *U* = 700.5, *r* = −0.17, *p* = 0.258; lymphocytes: *t*(45) *=* 0.90, *d* = 0.26, *p* = 0.374; monocytes: *t*(45) *=* *−*0.39, *d* = −0.12, *p* = 0.696) or at *t*_2_ (leukocytes: *U* = 723.5, *r* = −0.03, *p* = 0.853; lymphocytes: *t*(48) = 1.12, *d* *=* 0.32, *p* = 0.269; monocytes: *t*(48) *=* *−*0.23, *d* = −0.06, *p* = 0.822).

2 Samples of women with and without CM did not differ in the storage time of cryopreserved cells [*t*_0_: *U* = 710.50, *r* = 0.15, *p* = 0.285; *t*_2_: *t*(50) = −0.04, *d* = −0.01, *p* = 0.967].

The linear mixed effect models for the standard hemograms revealed that the blood composition did not differ between CM− and CM+ group but changed significantly from *t*_0_ to *t*_2_ in both groups (main effect Time; *p* < 0.001). Whereas the number of leukocytes in whole blood significantly decreased, the percentage of lymphocytes and monocytes in whole blood significantly increased from *t*_0_ to *t*_2_ in both groups.

### Mitochondrial respiration in intact PBMC

As previously described, mitochondrial respiration in intact PBMC was measured by high-resolution respirometry using a Substrate-Uncoupler-Inhibitor-Titration (SUIT) protocol on an air-calibrated O2k Oxygraph (Oroboros Instruments, Innsbruck, Austria) (Boeck et al., 2016, 2018a; Gumpp et al., 2020). Physiological respiration (*Routine* respiration) was first measured as basal mitochondrial oxygen consumption of intact PBMC. The addition of 0.5 *μ*l of the ATP synthase (Complex V)-inhibitor Oligomycin (5 mm stock; Sigma-Aldrich) induced *Leak* respiration, which compensates for proton leak, slippage, and cation cycling over the inner mitochondrial membrane (Pesta & Gnaiger, 2012). The difference between *Routine* respiration and *Leak* respiration represents the *ATP-turnover-*related respiration and reflects the oxygen consumption related to ATP production by the ATP synthase. Then the uncoupler FCCP (1 mm stock; Sigma-Aldrich) was titrated (first 1 *μ*l, followed by 0.5 *μ*l steps) to measure the *Uncoupled* respiration, which indicates the non-physiological maximal capacity of the respiratory chain that is not limited by the enzyme activity of ATP synthase. *Spare respiratory capacity* is calculated as the difference between *Uncoupled* respiration and *Routine* respiration and represents the additional capacity of the electron transport system (ETS) to produce ATP on top of the basal cellular energy demand. Thereafter, the addition of 1 *μ*l Complex I-inhibitor Rotenone (1 mm stock; Sigma-Aldrich) and 1 *μ*l Complex III-inhibitor Antimycin A (5 mm stock; Sigma-Aldrich) enables the measurement of the so-called *Residual oxygen consumption* (ROX). ROX estimates the cellular oxygen consumption linked to oxidative side reactions remaining after the complete blocking of the respiratory chain (Pesta & Gnaiger, 2012) as well as the technical background noise of the oxygraph. According to the manufacturer's recommendations (Pesta & Gnaiger, 2012), ROX was subtracted from all other values to evaluate the cellular oxygen consumption related to mitochondrial activity.

For further qualitative analysis, flux control ratios were calculated based on the measured respiratory values as internal normalization to control for cell size, cell morphology, and mitochondrial content (Pesta & Gnaiger, 2012): Routine Control Ratio (*Routine* respiration/*Uncoupled* respiration); Leak Control Ratio (*Leak* respiration/*Uncoupled* respiration); Net Routine Control Ratio (*ATP-turnover*-related respiration/*Uncoupled* respiration); Coupling Efficiency (*ATP-turnover-*related respiration/*Routine* respiration).

Samples were measured in duplicates and blinded concerning group assignment. Raw values of both oxygraph chambers were normalized for the number of living cells in the sample and averaged oxygen consumption rates were used for statistical analyses (see Table 2). After the experiment, 500 000 cells were shock-frozen in liquid nitrogen for the measurement of mitochondrial density.

### Spectrophotometric measurements of the mitochondrial density per cell

To quantify the density of the mitochondrial network per cell, it is the gold standard to measure the activity of the mitochondrial enzyme Citrate synthase (Larsen et al., 2012). Following respirometry, *Citrate-synthase* activity (CSA) was determined spectrophotometrically at 30 °C as previously described (Boeck et al., 2016; Eigentler et al., 2012; Gumpp et al., 2020; Karabatsiakis et al., 2014). Values from high-resolution respirometry were normalized for the intracellular density of mitochondria via division by the *Citrate-synthase* activity.

### Data analysis

All statistical analyses were conducted with R 3.6.1 (R Core Team, 2019). Student's *t* tests were used to test for differences between groups that were normally distributed with equal variances. Upon nonnormality and/or variance inhomogeneity, non-parametric Mann-Whitney *U* tests were used. Linear mixed effect models (using the R package *lme4*; Bates, Mächler, Bolker, & Walker, 2015) were used to investigate whether mitochondrial respiration and density (i) changed from *t*_0_ to *t*_2_ (main effect Time), (ii) differed between CM− and CM+ mothers (main effect CM group), and (iii) whether the changes over time were group-specific (CM group × Time interaction). As data were nested within participants due to repeated measures, random intercepts were modeled for each participant. Histograms were examined to ensure a sufficient normal distribution of model residuals. Models applied type-III squared sums. All reported analyses were performed two-tailed with the significance level set at *p* ≤ 0.05.

Subsequently, the nature of Time × CM group interactions was explored in detail using *post-hoc* tests (i.e. linear contrasts, using the *emmeans* package for R, Lenth, 2019). *p* values of the *post-hoc* tests to explore an interaction were adjusted with the Benjamini-Hochberg (false discovery rate, FDR) procedure (Benjamini & Hochberg, 1995). Duration of cell cryopreservation served as a covariate for the mitochondrial parameters as it affects mitochondrial functioning of cryopreserved cells (Keane, Calton, Cruzat, Soares, & Newsholme, 2015; Larsen et al., 2012; Nicholas et al., 2017). Samples of women with and without CM did not differ in duration of cell cryopreservation [*t*_0_: *U* = 710.50, *r* = 0.15, *p* = 0.285; *t*_2_: *t*(50) = −0.04, *d* = −0.01, *p* = 0.967; see Table 2].

## Results

**Fig. 1.**
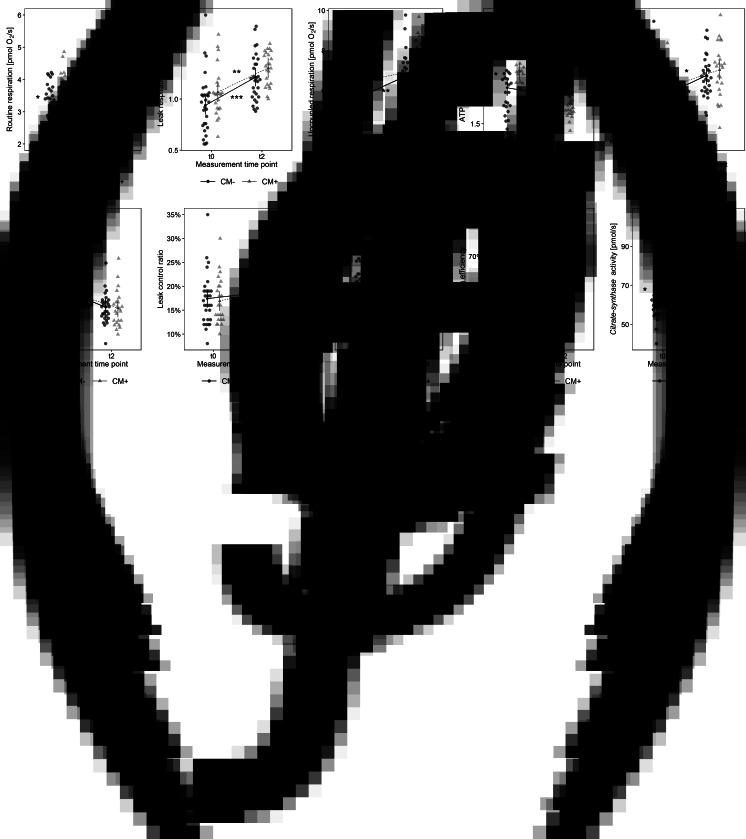
The change in mitochondrial respiration and density from 1 week postpartum (*t*_0_) to 1 year postpartum (*t*_2_) in *n* = 52 women with a history of childhood maltreatment (CM+, *n* = 23) and without (CM−, *n* = 29). Results of the linear mixed effect models are presented as predicted mean ± model-based 95% confidence intervals on the background of raw data. Mitochondrial respiration (*a*–*e*) was measured by high-resolution respirometry in pmol O_2_/s per Million living cells. Flux control ratios (*f*–*i*) were calculated based on the measured respiratory values. As a marker for mitochondrial density (mitochondrial biogenesis), *Citrate-synthase* activity in pmol/s per Million shock-frozen cells was spectrophotometrically measured. Significant post-hoc tests are denoted by *p < 0.050, **p < 0.010, ***p < 0.001.

The linear mixed effect models revealed significant CM group × Time interactions for *ATP-turnover-*related respiration (*p* = 0.048), CSA (*p* = 0.033), and for *Routine* respiration in trend (*p* = 0.075; Table 3). As displayed in Fig. 1, at *t*_0_, CM+ mothers exhibited significantly higher *Routine* (*p_FDR_* = 0.011; Fig. 1*a*), *ATP-turnover-*related respiration (*p_FDR_* = 0.016; Fig. 1*d*), and CSA (*p_FDR_* = 0.016; Fig. 1*j*) than CM- mothers, while these differences were not found at *t*_2_ (all *p_FDR_* > 0.500; Table 3). Correspondingly, the *ATP-turnover-*related respiration in CM+ mothers decreased significantly from *t*_0_ to *t*_2_ (*p*_FDR_ < 0.001; Table 3, Fig. 1*d*).

**Table 3.** Results of linear mixed effect models for mitochondrial respiration in women (*n* = 52)

Outcome	Predictor	*b* (s.e.)	95% CI (*b*)	*β*	*η*^2^	*F*	*p*	*Post-hoc* tests^1^		
								Compared groups	*b* (s.e.)	*p*
*Routine* respiration	Intercept	4.50 (0.28)	[3.96, 5.03]			260.77	<0.001***	CM− v. CM+ *t*_0_	0.43 (0.14)	0.011*
	CM	0.43 (0.14)	[0.16, 0.71]	0.38	0.049	9.53	0.003**	CM− *v*. CM+ *t*_2_	0.07 (0.14)	0.600
	Time	0.12 (0.14)	[−0.14, 0.38]	0.11	0.003	0.77	0.384	CM− *t*_0_ *v*. *t*_2_	0.12 (0.14)	0.511
	Duration of Cryopreservation	−1.19 × 10^−3^ (2.41 × 10^−4^)	[−1.65 × 10^−3^, −7.23 × 10^−4^]	−0.45	0.182	23.86	<0.001***	CM+ *t*_0_ *v*. *t*_2_	−0.24 (0.15)	0.220
	CM × Time	−0.36 (0.20)	[−0.74, 0.02]	−0.26	0.025	3.31	*0.075*			
	Overall model statistics: *F*(4,74.99) = 8.03, *p* < 0.001***, *R*^2^ = 0.249 (0.249), *σ*_ri_ = 0.000									
*Leak* respiration	Intercept	1.07 (0.14)	[0.81, 1.34]			60.63	<0.001***			
	CM	0.13 (0.07)	[−6.63 × ^−5^, 0.26]	0.24	0.038	3.72	*0.057*			
	Time	0.27 (0.06)	[0.16, 0.39]	0.51	0.251	20.49	<0.001***			
	Duration of Cryopreservation	−1.17 × 10^−4^ (1.20 × 10^−4^)	[−3.50 × 10^−4^, 1.14 × 10^−4^]	−0.09	0.008	0.93	0.337			
	CM × Time	−0.04 (0.09)	[−0.21, 0.13]	−0.06	0.002	0.19	0.662			
	Overall model statistics: *F*(4,75.37) = 9.18, *p* < 0.001***, *R*^2^ = 0.346 (0.246), *σ*_ri_ = 0.087									
*ATP-turnover-*related respiration	Intercept	3.43 (0.22)	[3.01, 3.86]			236.83	<0.001***	CM− *v*. CM+ *t*_0_	0.31 (0.11)	0.016*
	CM	0.31 (0.11)	[0.09, 0.52]	0.30	0.022	7.33	0.008**	CM− *v*. CM+ *t*_2_	−0.02 (0.11)	0.882
	Time	−0.15 (0.11)	[−0.36, 0.06]	−0.15	0.097	2.01	0.162	CM− *t*_0_ *v*. *t*_2_	−0.15 (0.11)	0.216
	Duration of Cryopreservation	−1.08 × 10^−3^ (1.93 × 10^−4^)	[−1.45 × 10^−3^, −7.08 × 10^−4^]	−0.46	0.206	30.77	<0.001***	CM+ *t*_0_ *v*. *t*_2_	−0.48 (0.12)	<0.001***
	CM × Time	−0.32 (0.16)	[−0.63, −0.02]	–0.27	0.027	4.10	0.048*			
	Overall model statistics: *F*(4,74.99) = 15.53, *p* < 0.001***, *R*^2^ = 0.381 (0.381), *σ*_ri_ = 0.000									
*Uncoupled* respiration	Intercept	7.58 (0.67)	[6.29, 8.88]			124.94	<0.001***	CM− *v*. CM+ *t*_0_	0.99 (0.34)	0.010*
	CM	0.99 (0.34)	[0.33, 1.65]	0.37	0.057	8.35	0.005**	CM− *v*. CM+ *t*_2_	0.32 (0.34)	0.349
	Time	1.20 (0.33)	[0.56, 1.84]	0.45	0.093	13.11	<0.001***	CM− *t*_0_ *v*. *t*_2_	1.20 (0.33)	0.003**
	Duration of Cryopreservation	−1.78 × 10^−3^ (5.87 × 10^−4^)	[−2.91 × 10^−3^, −6.48 × 10^−4^]	−0.28	0.071	9.03	0.004**	CM+ *t*_0_ *v*. *t*_2_	0.53 (0.36)	0.202
	CM × Time	−0.67 (0.48)	[−1.60, 0.26]	−0.21	0.015	1.92	0.171			
	Overall model statistics: *F*(4,74.99) = 6.24, *p* < 0.001***, *R*^2^ = 0.198 (0.198), *σ*_ri_ = 0.000									
*Spare respiratory capacity*	Intercept	3.09 (0.58)	[1.96, 4.22]			27.48	<0.001***			
	CM	0.56 (0.30)	[−0.02, 1.13]	0.24	0.030	3.49	*0.065*			
	Time	1.08 (0.29)	[0.52, 1.63]	0.47	0.149	14.07	<0.001***			
	Duration of Cryopreservation	−5.95 × 10^−4^ (5.10 × 10^−4^)	[−1.58 × 10^−3^, 3.89 × 10^−4^]	−0.11	0.011	1.34	0.252			
	CM × Time	−0.31 (0.42)	[−1.12, 0.50]	−0.11	0.004	0.54	0.465			
	Overall model statistics: *F*(4,74.99) = 5.65, *p* < 0.001***, *R*^2^ = 0.182 (0.182), *σ*_ri_ = 0.000									
*Citrate-synthase* activity	Intercept	49.58 (5.36)	[39.02, 59.95]			84.05	<0.001***	CM− *v*. CM+ *t*_0_	7.83 (2.69)	0.018*
	CM	7.83 (2.69)	[2.64, 13.02]	0.38	0.035	8.48	0.004**	CM− *v*. CM+ *t*_2_	−0.22 (2.67)	0.933
	Time	4.71 (2.53)	[−0.21, 9.68]	0.23	0.001	3.45	*0.068*	CM− *t*_0_ *v*. *t*_2_	4.71 (2.53)	0.137
	Duration of Cryopreservation	0.01 (0.01)	[0.003, 0.02]	0.25	0.059	6.26	0.015*	CM+ *t*_0_ *v*. *t*_2_	−3.34 (2.77)	0.310
	CM × Time	−8.05 (3.69)	[−15.28, −0.85]	−0.33	0.044	4.76	0.034*			
	Overall model statistics: *F*(4,75.14) = 4.58, *p* = 0.002**, *R*^2^ = 0.198 (0.154), *σ*_ri_ = 2.178									

*Note*. *p < 0.050, **p < 0.010, ***p < 0.001, two-tailed. Italic *p* values indicate a trend for significance (*p* < 0.100). All models are random intercept models (*σ*_ri_… standard deviation of random intercepts). Coefficients of determination (*R*^2^) present variance explanation of the total model (including random effects) and, in brackets, variance explanation by fixed effects (i.e. model predictors) only. CM− as a reference group.

1 Linear *post-hoc* tests were performed to describe the nature of the significant interaction effects. *p* Values were adjusted for multiple comparisons with the false discovery rate (FDR).

In addition, *Leak* respiration and *Spare respiratory capacity* did not differ between groups but increased significantly from *t*_0_ to *t*_2_ in both groups (*p* < 0.001; Table 3; Fig. 1*b* and 1*e*).

Furthermore, there were significant effects of CM group and Time on *Uncoupled* respiration, while the CM group × Time interaction was not significant (*p* = 0.171; Table 3). *Post-hoc* tests revealed that CM+ women had significantly higher *Uncoupled* respiration than CM− women at *t*_0_ (*p_FDR_* = 0.010), although this difference disappeared at *t*_2_ (*p*_FDR_ = 0.349), since *Uncoupled* respiration significantly increased from *t*_0_ to *t*_2_ in CM− women only (*p_FDR_* = 0.003; Fig. 1*c*).

We repeated the analyses with the respiration parameters normalized for CSA. As a result, any main effects of CM group and CM group × Time interaction effects diminished, whereas the effects of Time remained significant (see online Supplementary Table S2).

Analyzing the flux control ratios revealed, that Routine control ratio (*p* < 0.001), Net routine ratio (*p* < 0.001), and Coupling Efficiency (*p* < 0.001) significantly decreased from *t*_0_ to *t*_2_ in both groups (Table 4 and Fig. 1*f*–*i*). Besides, there were neither significant CM group × Time interactions nor significant main effects of CM group (Table 4).

**Table 4.** Results of linear mixed effect models for mitochondrial flux control ratios in women (*n* = 52)

Outcome	Predictor	*b* (s.e.)	95% CI (*b*)	*β*	*η*^2^	*F*	*p*	*Post-hoc* tests^1^
Routine control ratio (%)	Intercept	0.66 (0.06)	[0.55, 0.77]			137.32	<0.001***	
	CM	−0.02 (0.03)	[−0.07, 0.03]	−0.10	0.003	0.58	0.447	
	Time	−0.88 (0.03)	[−0.14, −0.04]	−0.42	0.161	12.22	<0.001***	
	Duration of Cryopreservation	−6.95 × 10^−5^ (4.86 × 10^−5^)	[−1.72 × 10^−4^, 2.77 × 10^−5^]	−0.14	0.019	2.00	0.162	
	CM × Time	0.02 (0.04)	[−0.05, 0.09]	0.07	0.002	0.22	0.640	
	Overall model statistics: *F*(4,75.30) = 6.56, *p* < 0.001***, *R*^2^ = 0.278 (0.190), *σ*_ri_ = 0.032							
Leak control ratio (%)	Intercept	0.14 (0.03)	[0.08, 0.20]			23.03	<0.001***	
	CM	−4.99 × 10^−3^ (0.01)	[−0.03, 0.02]	−0.05	<0.001	0.13	0.724	
	Time	0.01 (0.01)	[−0.01, 0.04]	0.12	0.040	0.92	0.340	
	Duration of Cryopreservation	2.95 × 10^−5^ (2.53 × 10^−5^)	[−2.09 × 10^−5^, 7.87 × 10^−5^]	0.12	0.015	1.33	0.252	
	CM × Time	0.01 (0.02)	[−0.02, 0.05]	0.10	0.005	0.43	0.517	
	Overall model statistics: *F*(4,75.45) = 1.70, *p* = 0.159, *R*^2^ = 0.207 (0.057), *σ*_ri_ = 0.020							
Net routine ratio (%)	Intercept	0.52 (0.04)	[0.44, 0.59]			184.53	<0.001***	
	CM	−0.02 (0.02)	[−0.05, 0.02]	−0.10	0.007	0.87	0.353	
	Time	−0.10 (0.02)	[−0.14, −0.07]	−0.58	0.355	34.04	<0.001***	
	Duration of Cryopreservation	−9.62 × 10^−5^ (3.29 × 10^−5^)	[−1.66 × 10^−4^, −3.04 × 10^−5^]	−0.23	0.054	8.38	0.005**	
	CM × Time	6.66 × 10^−3^ (0.03)	[−0.04, 0.06]	0.03	<0.001	0.07	0.796	
	Overall model statistics: *F*(4,75.18) = 21.22, *p* < 0.001***, *R*^2^ = 0.479 (0.442), *σ*_ri_ = 0.017							
Coupling efficiency (%)	Intercept	0.81 (0.03)	[0.74, 0.87]			544.37	<0.001***	
	CM	1.05 × 10^−3^ (0.02)	[−0.03, 0.03]	<0.01	0.002	0.004	0.949	
	Time	−0.08 (0.01)	[−0.11, −0.05]	−0.51	0.452	33.51	<0.001***	
	Duration of Cryopreservation	−9.24 × 10^−5^ (3.00 × 10^−5^)	[−1.51 × 10^−4^, −3.46 × 10^−5^]	−0.26	0.059	9.25	0.003**	
	CM × Time	−0.02 (0.02)	[−0.05, 0.02]	−0.09	0.005	0.73	0.396	
	Overall model statistics: *F*(4,75.77) = 27.06, *p* < 0.001***, *R*^2^ = 0.601 (0.448), *σ*_ri_ = 0.031							

*Note*. *p < 0.050, **p < 0.010, ***p < 0.001, two-tailed. All models are random intercept models (*σ*_ri_… standard deviation of random intercepts). Coefficients of determination (*R*^2^) present variance explanation of the total model (including random effects) and, in brackets, variance explanation by fixed effects (i.e. model predictors) only. CM− as a reference group.

1 Linear *post-hoc* tests were not performed as the CM × Time interactions were not significant.

## Discussion

So far, mitochondrial bioenergetics were mainly examined in cross-sectional studies without providing information about the temporal stability of findings. For the first time, we investigated longitudinal alterations in mitochondrial respiration and density in PBMC of women with and without a history of CM at shortly after parturition (*t*_0_) and 1 year after pregnancy (*t*_2_). Thereby, we questioned whether our previous observations of upregulated mitochondrial bioenergetic profiles in PBMC of postpartum women with CM (Boeck et al., 2016; Gumpp et al., 2020) remain stable over time or temporarily peak during phases of allostatic load, namely temporarily elevated inflammation at the postpartum period. Our results revealed that, independently of the women's CM experiences, the mitochondria of all women exhibited higher flux-control ratios at *t*_0_ as compared to *t*_2_. In contrast, the mitochondrial respiration parameters *Leak* respiration and *Spare respiratory capacity* increased from *t*_0_ to *t*_2_.

In healthy postpartum women, the immune system is stimulated by labor as well as delivery to increase the production of pro-inflammatory cytokines, resulting in an upregulation of inflammatory processes (Corwin et al., 2003; Maes et al., 2001). Across the next weeks to months, women recover from childbirth and inflammation regresses. With recovery, the women's HPA axis function, which is suppressed following delivery, normalizes and its hormones return to confine the inflammation (Mastorakos & Ilias, 2003).

In line with this, we found in our cohort a significant decrease of the white blood cell count (number of leukocytes in whole blood) from *t*_0_ to *t*_2_ in both groups indicating a decrease in inflammatory processes from *t*_0_ to *t*_2_. Leukocytes are mediators of inflammation and have a key role in host defense to injury. Increased white blood cell count has been associated with several adverse health conditions, like diabetes (Vozarova et al., 2002), coronary heart disease (Madjid & Fatemi, 2013), or a worse outcome in the general population (De Labry, Campion, Glynn, & Vokonas, 1990).

Further supporting this perspective, we found higher flux-control ratios shortly after parturition compared to one year later, indicating that the capacity of mitochondria in PBMC was temporarily enhanced in order to meet the increased energy demand. The decrease in the mitochondrial flux-control ratios and the corresponding increase of *spare respiratory capacity* from *t*_0_ to *t*_2_ could imply that at 1 year after parturition, a smaller proportion of the mitochondria's performance capacity is used for basal physiological activities. The increase in *spare respiratory capacity*, which represents the additional mitochondrial capacity to produce ATP on top of the basal cellular energy demand, might reflect a higher ability of PBMC to produce more ATP in times of increased energy demands to a second stimulus, e.g. an additional stressor or an infection.

Reference values from non-pregnant, non-stressed individuals are not yet available. However, our results indicate that at 1 year after parturition, the mitochondrial respiration and density and presumably also the physiological and immunological responses of the women are recovered to the state before pregnancy. The higher performance load on mitochondria in the period shortly after pregnancy might lead to wear and tear of the mitochondria themselves. Mitochondria are not only the main producers but also the main target of ROS (Lee & Wei, 2005), leading to less performance of ROS-damaged mitochondria. After 1 year, it might be that the mitochondrial ROS production is reduced, the ROS-induced mitochondrial damages are repaired, and/or new mitochondria are generated.

Concerning CM experiences of the women, our study further revealed that shortly after parturition (*t*_0_), PBMC of CM-affected women exhibited significantly higher mitochondrial respiration and density than those of women without CM. In detail, at *t*_0_, the basal physiological activity of mitochondria (i.e. *Routine* respiration), their energy (ATP) production (i.e. *ATP-turnover*-related respiration), maximal respiratory capacity (i.e. *Uncoupled* respiration), and intracellular density (measured as CSA) was higher in PBMC of women with CM history than of women without. Moreover, we found no differences in the flux-control ratios between women with and without CM at *t*_0_. This result pattern was already reported in a larger study cohort of the ‘My Childhood – Your Childhood’ study (Gumpp et al., 2020).

Repeating the analyses with the respiration parameters normalized for CSA, the significant CM group × time interactions as well as the significant CM group main effects diminished. Thus, the increased overall cellular respiratory activity observed in CM+ women shortly after parturition (*t*_0_) was likely due to an elevated mitochondrial density per cell. This higher amount of mitochondria per cell increases the total respiration capacity of PBMC, possibly to provide the immune system of CM+ mothers with additional energy after parturition (see Gumpp et al., 2020 for more detail). A previous study of ours showed that at 3 months postpartum, mitochondria of women with CM still exhibited higher basal mitochondrial activity and an increased ATP production compared to women without CM; however, their intracellular mitochondrial density was already reduced (Boeck et al., 2016). With the present study, we showed that at 1 year after parturition (*t*_2_), the group differences in mitochondrial density as well as in mitochondrial respiration between CM− and CM+ women vanished. Hence, a history of CM did no longer account for differences in mitochondrial function and density at *t*_2_. Altogether, these results suggest that in the months after parturition, there is a gradual reduction in mitochondrial density and subsequently in mitochondrial performance.

Conceivably, the postpartum course of mitochondrial bioenergetics is tightly linked to higher energy demand in the immune system of postpartum women with CM. There is growing evidence that early adversity is associated with larger acute stress-induced increases in inflammation (reviewed in Fagundes, Glaser, & Kiecolt-Glaser, 2013). Therefore, we speculate that women with and without CM do not significantly differ in their basal level of peripheral inflammatory markers but in their reactivity of immune cells to external as well as internal immunological challenges (such as stress, infections, or wound healing in the postpartum phase). Moreover, chronic and traumatic stress is associated with slower wound healing, probably due to an impaired immune regulation (Christian et al., 2006; Kiecolt-Glaser et al., 1995). Wound healing in CM-affected women might also be compromised or even delayed. The inflammatory response following delivery is elevated in women who previously suffered from depression, suggesting that depression is accompanied by a sensitization of the inflammatory response system (Maes et al., 2001). In line with these findings, our data suggest that the inflammatory response following delivery might also be elevated in women with a history of CM. It could be speculated that the mutual enhancement of mitochondrial activity and inflammation leads to a prolongation of inflammatory activity and slower healing in CM-affected individuals. Indeed, we provided evidence that increase in mitochondrial respiratory activity was related to the higher release of pro-inflammatory cytokines by PBMC of CM-affected women at 3 months postpartum (Boeck et al., 2016). These processes altogether present an additional energy demand for the immune system of CM-affected women that needs to be met by immunocellular mitochondria.

Altogether, we speculate that after giving birth, the immune cells of women without CM temporarily increase their mitochondrial performance as reflected by higher flux-control ratios compared to 1 year later. Women with CM, whose immune regulation is impaired, might not only need to increase their mitochondrial performance after parturition, but also their mitochondrial density. Consequently, inflammation and oxidative stress levels might also increase even stronger in women with CM. We postulate that, in women with CM history, the sensitization of the inflammatory response after delivery might explain the higher mitochondrial respiration and density in their early postpartum period. Along with the physiological recovery from childbirth, inflammation regresses and the energy demand of immune cells decreases, leading to a reduction in mitochondrial flux-control ratios in all women. Similarly, mitochondrial density in CM-affected women normalizes during recovery. Moreover, hormonal shifts occur during pregnancy and the postpartum period (reviewed in Hendrick, Altshuler, & Suri, 1998) as part of the physical recovery from childbirth and the preparation of the female body for nursing, including oxytocin and prolactin secretion contribute to mother-infant bonding and lactation regulation. Postpartum changes in oxytocin levels could possibly contribute to altered mitochondrial bioenergetics. Indeed, we recently reported a significant interaction between the severity of CM experiences and oxytocin on cellular oxygen consumption of PBMC at 3 months postpartum: in individuals with higher CM severity, higher oxytocin levels were associated with decreased basal mitochondrial respiration and ATP turnover (Boeck et al., 2018a).

Besides, normal human pregnancy dramatically affects the maternal HPA axis (reviewed in Lindsay & Nieman, 2005), resulting in progressive rises in glucocorticoids with advancing gestation. During the postpartum period, the HPA axis gradually recovers from its activated state during pregnancy (Jung et al., 2011; Mastorakos & Ilias, 2003).

CM is assumed to evoke a complex HPA-axis dysregulation (reviewed in Guilliams & Edwards, 2010; Tarullo & Gunnar, 2006), and there is evidence on bidirectional connections between glucocorticoids and the physiology and function of mitochondria (Lapp, Ahmed, Moore, & Hunter, 2019; Picard, Juster, & McEwen, 2014). Conceivably, pregnancy-related shifts in glucocorticoid levels might affect mitochondrial bioenergetics and could contribute to differences in mitochondrial respiration and density between CM− and CM+ women occurring at early postpartum and which normalize 1 year later. Recently, we could show a significant interaction between the severity of CM experiences and cortisol on cellular oxygen consumption of PBMC at 3 months postpartum: higher cortisol levels were associated with an increase in cellular oxygen consumption related to basal mitochondrial respiration and ATP turnover in individuals with higher CM severity (Boeck et al., 2018a). Additional studies are required to elucidate the relevance of post-pregnancy hormonal changes in the regulation of mitochondrial bioenergetics.

In sum, our results indicate that CM-related mitochondrial alterations could only be detectable under allostatic load, e.g. the immunological challenge of parturition and wound healing in our study, and may disappear later after birth, i.e. when the immune reaction eventually weakens and the acute energy requirement decreases. A higher allostatic load might lead to the observed differences in mitochondrial bioenergetics at *t*_0_ that are vanished by *t*_2_. However, women with a CM history might again differ in their response to subsequent additional stressors. Our study provides evidence that CM experiences influence the biological vulnerability for physical and mental stress later in the later life of affected individuals.

Further research is mandatory to replicate our findings, assess inflammatory markers to proof our suggested model, investigate the involvement of glucocorticoids in that context, and to investigate which external factors might explain the seen differences in mitochondrial function and density in immune cells. These factors should comprise psychosocial factors (e.g. ongoing psychosocial stress, traumatic stress, social support, mother-child interaction) as well as physiological stressors (e.g. sleep deprivation, physical activity, infections). Additionally, the influence of catecholamines in the context of mitochondrial bioenergetics in postpartum women needs further research as catecholamines were found to be increased during delivery. After labor, the catecholamine levels decreased to the levels during late pregnancy (Alehagen, Wijma, Lundberg, Melin, & Wijma, 2001). Investigating the link between mitochondria, inflammation, glucocorticoids, catecholamines, and depressive symptoms might further gain new insights also in the research of postpartum depression.

### Strengths

This is the first study to investigate mitochondrial bioenergetic profiles in a longitudinal design, measuring mitochondrial function and density repeatedly among the same women shortly with standardized and highly sensitive methods. We additionally assessed the activity of the mitochondrial enzyme Citrate synthase to consider the density of the intracellular mitochondrial network as an important marker for peripheral mitochondrial alterations. Furthermore, we included standard hemograms in our study to correct the possible influences of immunological alterations in the PBMC subset composition following pregnancy or parturition.

### Limitations

To generalize our findings and investigate whether CM-related alterations in mitochondrial bioenergetics become evident specifically under allostatic load, additional research is needed among men and women with concurrent physical or psychosocial stress. The mitochondrial bioenergetic profile was measured in cryopreserved cells and cryopreservation *per se* has an impact on the quantitative measures related to oxygen consumption rates (Gumpp et al., 2021). Although storage time of cryopreserved cells was statistically considered, we can thus not exclude that freezing procedure and cryopreservation might have differentially affected the immune cells of individuals with and without CM.

Importantly, PBMC comprise different proportions of immune cell subsets (i.e. lymphocytes, monocytes, and dendritic cells), which differ in mitochondrial respiration and density (Chacko et al., 2013; Kramer, Ravi, Chacko, Johnson, & Darley-Usmar, 2014). In this study, CM was not associated with the proportion of monocytes and lymphocytes in fresh whole blood. Furthermore, in a study with women at 3 months postpartum, the PBMC subset composition within thawed samples did not differ between women with and without CM history (Boeck et al., 2018b). Due to the study design and logistical reasons in the handling of blood samples in the maternity ward 24 h/day, it was not possible to directly measure inflammatory parameters at *t*_0_ and therefore it was also not done at *t*_2_. Furthermore, we were not able to collect data on dietary habits in the investigated sample which might attenuate or reduce inflammation and antioxidant buffer systems besides the effects of CM. However, this aspect of the modulatory influence of diet is highly interesting and relevant, and should be investigated in future studies. Another limitation is the small sample size of the study cohort, restricting its statistical power. The generalizability of our findings might be limited to the characteristics of the study cohort, i.e. a community sample of healthy women of European origin, all living in a partnership with a relatively high socioeconomic status. Our cohort consisted of healthy women who reported relatively low levels of CM load, which might mask some effects of more severe CM experiences and associated clinical outcomes on mitochondrial bioenergetics. Furthermore, the medication during (e.g. epidural analgesia) and after parturition (e.g. analgesics) might have an effect on the measured mitochondrial function and density at *t*_0_.

## Conclusion

Mitochondrial bioenergetic profiles of peripheral immune cells in healthy women changed across the first year after giving birth, indicating an adaptation of the mitochondrial network to environmental factors and challenges. Shortly after parturition, maternal CM exposure was linked to a higher mitochondrial density and, as a result, higher mitochondrial respiration per cell. In contrast, at 1 year postpartum, the CM-related alterations in mitochondrial function and density vanished. This observation suggests that CM-related alterations in the energy production of PBMC might only manifest in conditions of allostatic load such as the immunological challenges of parturition. To further promote knowledge in this field, CM should be investigated in non-pregnant individuals using a longitudinal design. We speculate that the biological effects of psychological stress might be enhanced during periods of inflammation. According to the allostatic load model, chronic stress conditions impart vulnerability to dysregulated responses, especially in situations with novel or additional stressors. Thus, impaired allostatic adaptation processes might be the pathway through which CM elevates the risk for poor mental and physical health throughout life.

## Supplementary material

For supplementary material accompanying this paper visit https://doi.org/10.1017/S0033291722000411. click here to view supplementary material
