# Supplementary material for: Investigating mitochondrial bioenergetics in peripheral blood mononuclear cells of women with childhood maltreatment from post-parturition period to one-year follow-up
Source: Psychol Med. 2022 Mar 21;53(9):3793–804. doi: 10.1017/S0033291722000411 (PMC10317795; doi:10.1017/S0033291722000411)
Supplement: Supplementary file 1 [file S0033291722000411sup001.docx]

**Supplementary information**

**Investigating Mitochondrial Bioenergetics in Peripheral Blood Mononuclear Cells of Women With Childhood Maltreatment From Post-Parturition Period to One-Year Follow-up**

Anja M. Gumpp^1^, Alexander Behnke^1^, Laura Ramo-Fernández^1^, Peter Radermacher^2^, Harald Gündel^3^, Ute Ziegenhain^4^, Alexander Karabatsiakis^1,5 *^, Iris-Tatjana Kolassa^1 *^

* Authors share authorship in the senior position

**Supplementary Figures**

**
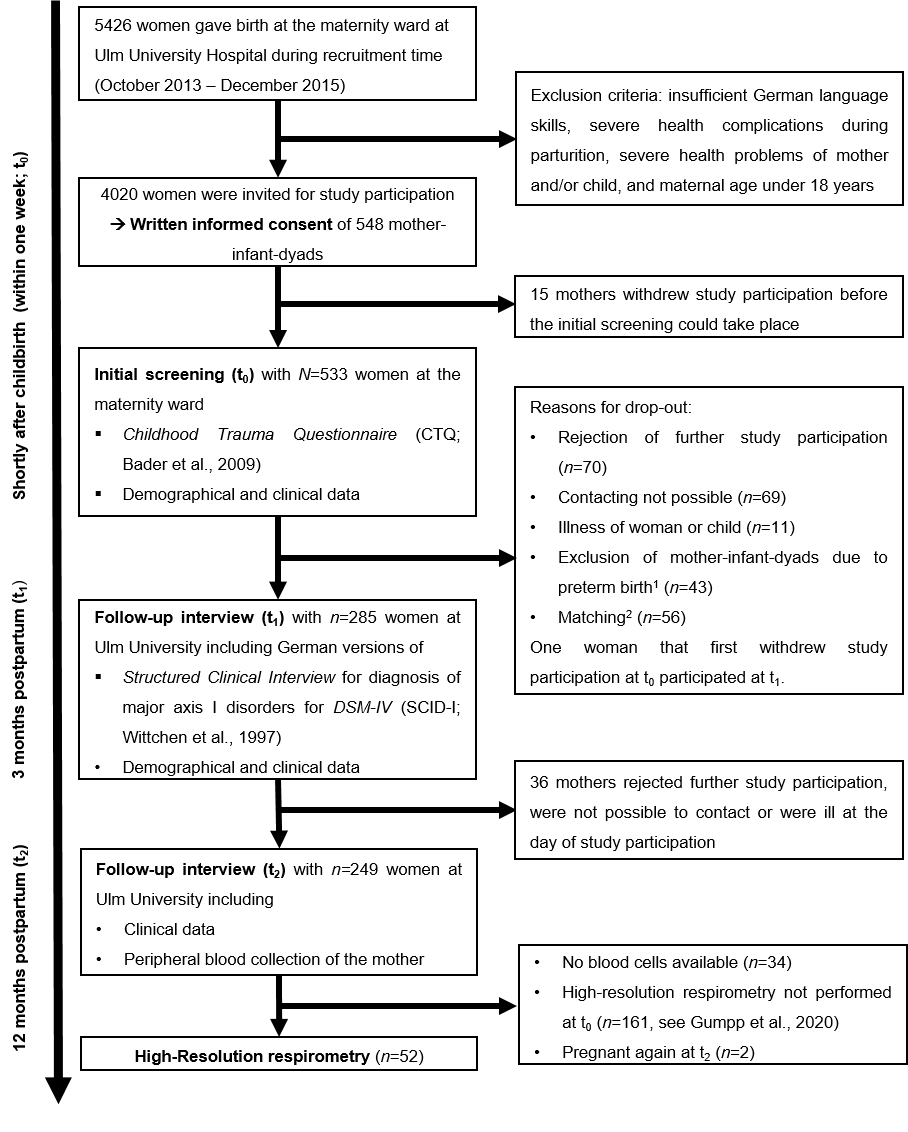
**

**Figure S1:** Flow chart representing the procedures of recruitment, characterization and analyses of the study cohort in the project “My Childhood – Your Childhood”.

^1^ Another main focus of the overall project included hypotheses concerning the effects of maternal CM on the development of the child. For the purpose of testing these hypotheses in the first place, the exclusion of preterm births was chosen as further exclusion criteria at t_1_. ^2^ All women were categorized at t_0_ in two groups due to established CTQ cut-off criteria (Bernstein and Fink, 1998): 1) Women without any childhood maltreatment (CM) experiences (CM-) and 2) Women with at least mild to severe CM experiences (CM+). All CM+ women were contacted to participate at t_1_. CM- women were matched to the at t_1_ participating CM+ women according to the maternal age and their socioeconomic status. Thus, *n*=56 CM- women were not contacted at t_1_ due to this matching process.

**Supplementary tables**

**Table S1**: Biological raw data of the women at t_0_ and t_2_ (*n*=52)

|  | **t_0_ (*n*=52)** | | **t_2_ (*n*=52)** | |
| --- | --- | --- | --- | --- |
|  | **CM- group**  (*n*=29)  *Range*  *(Min – Max)* | **CM+ group**  (*n*=23)  *Range*  *(Min – Max)* | **CM- group**  (*n*=29)  *Range*  *(Min – Max)* | **CM+ group**  (*n*=23)  *Range*  *(Min – Max)* |
| **Primary mitochondrial respiration parameters (pmol O_2_/sec per Mio cells)** | | | | |
| Routine respiration | 2.20 – 4.22 | 2.50 – 4.86 | 2.11 – 4.62 | 2.71 – 3.83 |
| Leak respiration | 0.56 – 1.82 | 0.63 – 1.63 | 0.88 – 1.71 | 1.00 – 1.54 |
| ATP-turnover-related respiration | 1.40 – 3.19 | 1.71 – 3.35 | 1.12 – 3.54 | 1.37 – 2.52 |
| Uncoupled respiration | 3.32 – 9.56 | 4.41 – 8.64 | 3.97 – 9.77 | 3.96 – 9.68 |
| Spare respiratory capacity | 0.48 – 5.85 | 0.92 – 4.90 | 1.74 – 5.46 | 1.19 – 6.12 |
| Residual oxygen consumption (ROX) | 0 – 0.70 | 0 – 0.61 | 0.01 – 0.24 | 0.05 – 0.26 |
| **Mitochondrial density (pmol/sec per Mio cells)** | | | | |
| *Citrate-synthase* activity | 40.13 – 79.96 | 54.84 – 106.31 | 54.53 – 94.36 | 57.90 – 95.28 |
| **Flux Control Ratios (%)** |  |  |  |  |
| Routine control ratio | 39 – 89 | 39 – 82 | 33 – 68 | 37 – 70 |
| Leak control ratio | 8 – 35 | 10 – 30 | 11 – 29 | 11 – 28 |
| Net routine ratio | 24 – 68 | 27 – 59 | 18 – 40 | 22 – 42 |
| Coupling efficiency | 56 – 81 | 57 – 80 | 47 – 77 | 51 – 69 |

Primary mitochondrial respiration parameters are presented corrected for Residual oxygen consumption (ROX). CM=Childhood maltreatment. CM+=Women with at least mild to severe CM experiences. CM-=Women without CM experiences

**Table S2.** Results of linear mixed effect models for mitochondrial respiration normalized for *citrate-synthase activity* (CSA) in women (*n*=52)

| **Outcome** | **Predictor** | ***b* (*SE*)** | **95% CI (*b*)** | **β** | **η^2^** | ***F*** | ***p*** | **Post Hoc Tests** ^a^ | | |
| --- | --- | --- | --- | --- | --- | --- | --- | --- | --- | --- |
|  |  |  |  |  |  |  |  |  |  |  |
| **Routine respiration normalized for CSA** | Intercept | 0.08 (3.94*10^-3^) | [0.07, 0.09] |  |  | 407.48 | <0.001^***^ |  |  |  |
|  | CM | 9.35*10^-4^ (2.01*10^-3^) | [-2.94*10^-3^, 4.81*10^-3^] | 0.05 | 0.009 | 0.22 | 0.643 |  |  |  |
|  | Time | -2.23*10^-3^ (1.94*10^-3^) | [-5.97*10^-3^, 1.51*10^-3^] | -0.12 | 0.015 | 1.32 | 0.256 |  |  |  |
|  | Duration of Cryopreservation | -2.61*10^-5^ (3.44*10^-6^) | [-3.28*10^-5^, -1.95*10^-5^] | -0.61 | 0.368 | 56.56 | <0.001^***^ |  |  |  |
|  | CM ⨯ Time | 8.28*10^-4^ (2.83*10^-3^) | [-4.64*10^-3^, 6.30*10^-3^] | 0.04 | 0.001 | 0.09 | 0.771 |  |  |  |
|  | Overall model statistics: *F*(4,74.99) = 17.00, *p* < 0.001^***^, R² = 0.404 (0.404), σ_ri_ = 0.000 | | | | | | |  |  |  |
| **Leak respiration normalized for CSA** | Intercept | 0.02 (2.15*10^-3^) | [0.02, 0.02] |  |  | 84.16 | <0.001^***^ |  |  |  |
|  | CM | 3.14*10^-4^ (1.05*10^-3^) | [-1.71*10^-3^, 2.34*10^-3^] | 0.04 | 0.016 | 0.09 | 0.765 |  |  |  |
|  | Time | 3.00*10^-3^ (9.38*10^-4^) | [1.16*10^-3^, 4.82*10^-3^] | 0.37 | 0.236 | 10.19 | 0.002^**^ |  |  |  |
|  | Duration of Cryopreservation | -4.31*10^-6^ (1.88*10^-6^) | [-8.04*10^-6^, -6.57*10^-7^] | -0.22 | 0.058 | 5.10 | 0.027^*^ |  |  |  |
|  | CM ⨯ Time | 1.25*10^-3^ (1.36*10^-3^) | [-1.40*10^-3^, 3.92*10^-3^] | 0.13 | 0.010 | 0.85 | 0.361 |  |  |  |
|  | Overall model statistics: *F*(4,75.44) = 7.03, *p* < 0.001^***^, R² = 0.326 (0.200), σ_ri_ = 0.001 | | | | | | |  |  |  |
| **ATPturn-over-related respiration normalized for CSA** | Intercept | 0.06 (3.26*10^-3^) | [0.05, 0.07] |  |  | 344.88 | <0.001^***^ |  |  |  |
|  | CM | 5.04*10^-4^ (1.63*10^-3^) | [-2.64*10^-3^, 3.65*10^-3^] | 0.03 | 0.001 | 0.10 | 0.757 |  |  |  |
|  | Time | -5.21*10^-3^ (1.52*10^-3^) | [-8.23*10^-3^, -2.27*10^-3^] | -0.32 | 0.192 | 11.71 | 0.001^**^ |  |  |  |
|  | Duration of Cryopreservation | -2.24*10^-5^ (2.85*10^-6^) | [-2.84*10^-5^, -1.67*10^-5^] | -0.58 | 0.400 | 60.57 | <0.001^***^ |  |  |  |
|  | CM ⨯ Time | -3.12*10^-4^ (2.22*10^-3^) | [-4.63*10^-3^, 4.05*10^-3^] | -0.02 | <0.001 | 0.02 | 0.889 |  |  |  |
|  | Overall model statistics: *F*(4,75.18) = 27.54, *p* < 0.001^***^, R² = 0.553 (0.521), σ_ri_ = 0.002 | | | | | | |  |  |  |
| **Uncoupled respiration normalized for CSA** | Intercept | 0.14 (0.01) | [0.12, 0.16] |  |  | 169.71 | <0.001^***^ |  |  |  |
|  | CM | 4.51*10^-3^ (5.29*10^-3^) | [-5.70*10^-3^, 0.01] | 0.11 | 0.019 | 0.73 | 0.396 |  |  |  |
|  | Time | 0.01 (5.10*10^-3^) | [1.73*10^-3^, 0.02] | 0.28 | 0.093 | 5.14 | 0.027^*^ |  |  |  |
|  | Duration of Cryopreservation | -4.16*10^-5^ (9.06*10^-6^) | [-5.91*10^-5^, -2.41*10^-5^] | -0.43 | 0.175 | 20.66 | <0.001^***^ |  |  |  |
|  | CM ⨯ Time | 1.32*10^-3^ (7.46*10^-3^) | [-0.01, 0.02] | 0.03 | <0.001 | 0.03 | 0.860 |  |  |  |
|  | Overall model statistics: *F*(4,74.99) = 6.50, *p* < 0.001^***^, R² = 0.206 (0.206), σ_ri_ = 0.000 | | | | | | |  |  |  |
| **Spare respiratory capacity normalized for CSA** | Intercept | 0.06 (9.20*10^-3^) | [0.04, 0.07] |  |  | 36.58 | <0.001^***^ |  |  |  |
|  | CM | 3.55*10^-3^ (4.69*10^-3^) | [-5.50*10^-3^, 0.01] | 0.10 | 0.013 | 0.57 | 0.451 |  |  |  |
|  | Time | 0.01 (4.52*10^-3^) | [5.00*10^-3^, 0.02] | 0.39 | 0.146 | 9.20 | 0.004^**^ |  |  |  |
|  | Duration of Cryopreservation | -1.54*10^-5^ (8.03*10^-6^) | [-3.09*10^-5^, 8.84*10^-8^] | -0.18 | 0.036 | 3.61 | 0.062 |  |  |  |
|  | CM ⨯ Time | 5.18*10^-4^ (6.61*10^-3^) | [-0.01, 0.01] | 0.01 | <0.001 | 0.01 | 0.938 |  |  |  |
|  | Overall model statistics: *F*(4,74.99) = 4.63, *p* = 0.002^**^, R² = 0.155 (0.155), σ_ri_ = 0.000 | | | | | | |  |  |  |

*Note*: ^*^ *p* < 0.050, ^**^ *p* < 0.010, ^***^ *p* < 0.001, two-tailed. All models are random intercept models (σ_ri_… standard deviation of random intercepts). Coefficients of determination (R²) present variance explanation of the total model (including random effects) and, in brackets, variance explanation by fixed effects (i.e. model predictors) only. CM- as reference group.

^a^ Linear post-hoc tests were not performed as the CM ⨯ Time interactions were not significant.

**Supplementary references**

Bader K, Hänny C, Schäfer V, Neuckel A, Kuhl C (2009) Childhood Trauma Questionnaire – Psychometrische Eigenschaften einer deutschsprachigen Version. *Zeitschrift für Klinische Psychologie und Psychotherapie* 38, 223–230.

Bernstein D, Fink L (1998) Manual for the childhood trauma questionnaire. *New York: The Psychological Corporation*.

Gumpp AM, Boeck C, Behnke A, Bach AM, Ramo-Fernández L, Welz T, Gündel H, Kolassa I-T, Karabatsiakis A (2020) Childhood maltreatment is associated with changes in mitochondrial bioenergetics in maternal, but not in neonatal immune cells. *Proceedings of the National Academy of Sciences* 117, 24778–24784.

Wittchen H-U, Zaudig M, Fydrich T (1997) *SKID. Strukturiertes Klinisches Interview für DSM-IV.* Göttingen: Hogrefe.
